# Interdisciplinary Cardiovascular and Thoracic Surgery—2024 Reviewers

**DOI:** 10.1093/icvts/ivaf074

**Published:** 2025-05-12

On behalf of EACTS and EBCP, the Editor-in-Chief wishes to thank the following colleagues who have voluntarily given their valued time and effort to reviewing papers for ICVTS in 2024.

Mathias H. Aazami

Mohammed Nabil Abd Aljawad

Mohamed Abdel Bary

Moslem Abdelghafar

Amr Abdellateef

Hysam Elsyead Abdelmohty

Sami Aftab Abdul

Shinichiro Abe

Sosei Abe

Karen Beatrix Abeln

Djamila Abjigitova

Moustafa Farouk Aboollo

Anas Aboud

Youssef Fady Abouelela

Walid Abu Arab

Decio Cavalet Soares Abuchaim

Srilakshmi M. Adhyapak

Pankaj Aggarwal

Melek Ağkoç

Andrea Agostinelli

Apostolos Agrafiotis

Deepi Goyal Agrawal

Sunil Agrawal

Wolfgang William Schmidt Aguiar

Kamran Ahmadov

Ahmed Ahmed

Clemens Aigner

Mohammadhosein Akhlaghpasand

Zohair Al Halees

Ahmed Abdul Wahab Al-Bulushi

Alireza Alizadeh Ghavidel

Hani N. Alkattan

Saif Sami Almudhaffar

Safak Alpat

Nelson Alphonso

Obada Ahmad Alqudah

Bahaaldin Alsoufi

Peter Alston

Salah Eldien Altarabsheh

Mohammed Al-Tawil

Antonio Alvarez

Andrea Amabile

Marcello Carlo Ambrogi

Armin Amirian

Kevin Ruixuan An

Venkatesa Kumar Anakaputhur Rajan

Robert H. Anderson

Martin Andreas

Claudio Andreetti

Gianni D. Angelini

Philipp Angleitner

Amedeo Anselmi

Filippo Antonacci

Juan L. Anton-Pacheco

Zinar Apaydın

Muhammad Refaat Arab

Bardia Arabkhani

Amr A. Arafat

Akihito Arai

Mamoru Arakawa

Beatrice Aramini

Claudia Arenz

Olgun Kadir Aribaş

Rawa Arif

Arian Arjomandi Rad

Xavier Armario

Ahmet Arnaz

Zsuzsanna Arnold

Rakesh Christopher Arora

Sinan Arsan

Gokhan Arslan

Panagiotis Artemiou

Krishnasamy Arunkumar

Tohru Asai

Mitsuru Asano

Irene Asouhidou

Laura Asta

Ahmed Attia

Rizwan Q. Attia

John Augoustides

Daniel Auinger

Nicholas Austine

Ahmed K. Awad

Hüseyin Ayhan

Saima Azam

Mahesh B. Babu

Frank A. Baciewicz Jr.

Catalin Constantin Badiu

Nikolaos G. Baikoussis

Robert Balan

Ko Bando

Toru Bando

Annalisa Barbarossa

Gábor Bari

Mohammad A. Bashir

Adrian A.B. Bauer

Christophe Baufreton

Yusuf Bayrak

J.F. Matthias Bechtel

Erik Beckmann

Benoît Bédat

Jose Belda-Sanchis

Reda Belhaj Soulami

Hannah Rosemary Bellsham-Revell

John R. Benfield

Mikolaj Berezowski

Tim Berger

Rouven Berndt

Alexander M. Bernhardt

Ignacio Berra

Luca Bertoglio

Pietro Bertoglio

Luca Bertolaccini

Anne Beukers

Christina Beushausen

Branislav Bezak

Eric Bezon

Amit Bhargava

Fausto Biancari

Giacomo Bianchi

Benoit Jacques Bibas

Giuseppe Biondi-Zoccai

Arvind Kumar Bishnoi

Sabine Bleiziffer

Dmitry Bobylev

Wolfgang Boettcher

Nikolaos Bonaros

Johannes Bonatti

Stefano Bongiolatti

Andreas A. Böning

Michael A. Borger

Petre Vlah-Horea Botianu

Ilies Bouabdallah

Thierry Bove

Roland R. Brandt

Andrew Brazier

Denis Breshenkov

Pierre-Yves Brichon

Alessandro Brunelli

Vito Domenico Bruno

Hans Ulrich Burger

Nicola Buzzatti

Reda Bzikha

Marcin Marek Cackowski

Lucio Cagini

Zhixiang Cai

Antonio Maria Calafiore

Tulio Caldonazo

Alessio Campisi

Giuseppe Cardillo

Paulo F.G. Cardoso

Manuel Carnero-Alcazar

Matthew James Carr

Thierry Carrel

Angelo Carretta

Monica Casiraghi

Javier G. Castillo

Thierry Caus

David J. Chambers

Stephanie Chan

Andrew C. Chatzis

Cai Cheng

Yeung-Leung Cheng

Giovanni Alfonso Chiariello

Jong Bum Choi

Torsten Christ

Paola Ciriaco

Julie Cleuziou

Stephane Collaud

Hannah Copeland

Ana Rita Costa

Polina Danchenko

Mark H.D. Danton

Hiroshi Date

Piroze Davierwala

Giulia De Iaco

Laurent De Kerchove

Erik R. de Loos

Luca Salvatore De Santo

Georges Decker

Michelle Demetres

Manan Himanshu Desai

Shriprasad R. Deshpande

Gabriele Di Giammarco

Luca Di Marco

Ricardo R. Dias

Vanessa Diaz-Ravetllat

Chris Dickhoff

Zara Dietze

Arnaldo Dimagli

Ioannis Dimarakis

Wade Dimitri

Hongdou Ding

Christian Dinges

Philipp Discher

Aabha Divya

Torsten Doenst

Daniel-Sebastian Dohle

Esra Dönmez

Mario Giovanni Gerardo D'oria

Dimitrios Dougenis

Michael Downey

Paula Duarte D'ambrosio

Ryaan El-Andari

Sitaram Emani

Sandy Engelhardt

Florian Karl Enzmann

Giampiero Esposito

Anthony L. Estrera

David Faraoni

Paul Fedak

Francesco Formica

Stephen Edward Fremes

Örjan Friberg

Takuya Fujikawa

Ikuo Fukuda

Michele Gallo

Alejandro García-Pérez

Ovidio A. García-Villarreal

Jose M. Garrido

Cengiz Gebitekin

Marco Gemelli

Borut Gersak

Evaldas Girdauskas

Brian E. Glenville

Motohiko Goda

Can Gollmann-Tepekoylu

David Gomez de Antonio

Michel Gonzalez

Arun Gopalakrishnan

Roman Gottardi

Daniel A. Grandmougin

Paulo Gregorio

William Grossi

Paul F. Gründeman

Francesco Guerrera

Jeffrey A. Hagen

Christoph M. Haller

Claude E. Hanet

Jan Hinnerk Hansen

Amer Harky

Wael Hassanein

Hoda Hatoum

Claudia Heilmann

Khosro Hekmat

Israel Hernandez Ramirez

Eugene Hessel. II

Samuel Heuts

Tomas Holubec

Amir-Reza Hosseinpour

Hung-Lung Hsu

Shu-Chien Huang

Nabil Hussein

Ho Young Hwang

Mohsen Ibrahim

Yasunori Iida

Ilkka Ilonen

Teruhiko Imamura

Yoshito Inoue

Hironori Ishibashi

Fabio Ius

Krishna S. Iyer

Michael Jacobs

Katarzyna Januszewska

Omar A. Jarral

Marek Jasinski

Richard A. Jonas

Alberto Jorge Monteiro Dela Vega

Asher George Joseph

Alexander Kadner

Klaus Kaier

Satoshi Kainuma

Larry R. Kaiser

Klaus Kallenbach

Roya Kamali

Markus Kamler

Fikret Kanat

Emmanouil Ioannis Kapetanakis

Tevfik Kaplan

Tom R. Karl

Alexander Emil Kaspersen

Riken Kawachi

Michal J. Kawczynski

Aditya K. Kaza

W. Brent Keeling

Suresh Keshavamurthy

Krishna Khargi

Takashi Kido

Doosang Kim

Naoyuki Kimura

Michel Kindo

Bilal Haneef Kirmani

Eiri Kisamori

Olga N. Kislitsina

Christoph Knosalla

Alfred Kocher

Mladen J. Kocica

Maciej K. Kolowca

Igor E. Konstantinov

Tomislav Kopjar

Andre Korshin

Christophoros Kotoulas

Bartosz Kubisa

Hiroshi Kubota

Takashi Kunihara

Savvas Lampridis

Giovanni Landoni

Christopher Lau

Kyongjune Benjamin Lee

Eric J. Lehr

Miia L. Lehtinen

Mario Lescan

Antonio Lio

Huan Liu

Kevin Lobdell

Ana Lopez-Marco

Jose Lopez-Menendez

Roberto Lorusso

Heyman Luckraz

Arpad Lux

Wei-Guo Ma

Silvia Mariani

Mateo Marin-Cuartas

Luis C. Maroto

Thomas Martens

Klaus Matschke

Hitoshi Matsuda

Haruhisa Matsuguma

Nora Mayer

Massimiliano Meineri

Vanessa Menezes

Ari Mennander

Olaf Mercier

Carlos A. Mestres

Milan Milojevic

Kimito Minami

Kenji Minatoya

Eleonora Maddalena Minerva

Satyajeet Misra

Emilio Monguió

Nadejda Monsefi

Makoto Mori

Akimasa Morisaki

Selim Mosbahi

Marco Moscarelli

Giacomo Murana

Takashi Murashita

Felix Naegele

Pradeep Narayan

Eugenio Neri

Nuria M. Novoa

Vasileios Ntinopoulos

Maria Nucera

Suguru Ohira

Meinoshin Okumura

Francesco Onorati

Aung Oo

Leonardo Paim

Matthew S. Panagiotou

Nestoras Papadopoulos

Nikolaos A. Papakonstantinou

Vassil Papantchev

Haralabos Parissis

Kay-Hyun Park

Kartik Patel

Alexandro Patirelis

Jonas Pausch

Syed Murfad Peer

Louis P. Perrault

René Horsleben Petersen

Sven Peterss

George Petrov

Michele Piazza

Giulia Piccone

Gabriele Piffaretti

Antonios Pitsis

Leonard Pitts

Francesco Pollari

Leo Pölzl

Shi Sum Poon

Evgenij V. Potapov

Alberto Guido Pozzoli

Elena Prisciandaro

Pallavi Purwar

Tobias Pustjens

Mohamed Rahouma

Karthik Vaidyanathan Ramakrishnan

Madhuri Rao

Adeel Ur Rehman

David C. Reineke

Albert Rodríguez-Fuster

Maria Grazia Romeo

Sonia Ronchey

Antonino S. Rubino

Bartosz Rylski

Tomoki Sakata

Mohamed Salama

Sigrid Sandner

Elena Sandoval Martinez

Giuseppe Santarpino

Joao Santos Silva

Nishant Saran

Ulrik Sartipy

Milton Saute

Noriyoshi Sawabata

Paolo Scanagatta

Thomas Schachner

Hartzell V. Schaff

Simon Schalla

Stefano Schena

David Schibilsky

Jürg Schmidli

Paul Schoenhogen

Richard B. Schuessler

Davorin Sef

Burkhardt Seifert

Pierre Sentenac

Michael John Shackcloth

Mohammad Behgam Shadmehr

Norihiko Shiiya

Tsuyoshi Shoji

Jeffrey H. Shuhaiber

Serge Sicouri

Pranava Sinha

Thanos Sioris

Freyja Maria Smolle-Jüttner

Stefan Sponholz

Mila Stajevic

Lukas Stastny

Desiree Steimer

Yukiharu Sugimura

Staffan Svenmarker

Ben M. Swinkels

Federico Tacconi

Tohru Takaseya

Hiroshi Tanaka

Sara Tenconi

Stefan Thelin

Matthias Thielmann

Matthieu Thumerel

Alper Toker

Masayoshi Tokoro

Anton Tomsic

Theis Tonessen

Aybala Tongut

Beatrice Trabalza Marinucci

Nikolay O. Travin

Marko Ivan Turina

Akif Turna

Naomichi Uchida

Murat Ugurlucan

Daniel A. Valdivia

Arnaud Van Linden

Paul Van Schil

Stijn Vanstraelen

Praveen K. Varma

Bart Josephus Johannes Velders

Tiago R. Velho

Luigi Ventura

Peter Verbrugghe

Dominique Vervoort

Vibeke Videm

Eric Vinck

Konstantin Von Aspern

Ulrich Otto Von Oppell

Kerem M. Vural

Alexander Wahba

Isaac Wamala

Ryuichi Waseda

Katrin Welcker

Bernhard Winkler

David S. Winlaw

Dong Xie

Joseph Zacharias

Edoardo Zancanaro

Francesco Zaraca

Alicja Zientara

